# The influence of physical activity on risk of cardiovascular disease in people who are obese but metabolically healthy

**DOI:** 10.1371/journal.pone.0185127

**Published:** 2017-09-26

**Authors:** Shinje Moon, Chang-Myung Oh, Moon-Ki Choi, Yoo-Kyung Park, Sukyung Chun, Minkyung Choi, Jae Myung Yu, Hyung Joon Yoo

**Affiliations:** 1 Division of Endocrinology and Metabolism, Hallym University College of Medicine, Chuncheon, South Korea; 2 Division of Endocrinology and Metabolism, CHA Bundang Medical Center, School of Medicine CHA University, Pangyo, Seongnam, South Korea; Universitat de les Illes Balears, SPAIN

## Abstract

The metabolic outcomes of metabolically healthy obesity (MHO) remain controversial. The aim of the present study was to determine the effect of physical activity on the cardiovascular disease (CVD) outcomes of MHO. The study included participants who were followed for 10 years and recruited from the Korean Genome and Epidemiology Study (KoGES), a population-based cohort study. Participants with previously recorded CVDs or cancer, or who had received steroids or anticoagulants at baseline were excluded. A total of 8144 participants (3,942 men and 4,202 women) fulfilled inclusion criteria. In a multivariate Cox regression model adjusted for age and sex, MHO participants were not at elevated risk of CVD compared with their metabolically healthy non-obese (MHNO) counterparts (HR, 1.28; 95% CI, 0.96–1.71), although both the non-obese (HR, 1.50; 95% CI, 1.19–1.90) and obese (HR, 1.85; 95% CI, 1.48–2.30) participants with metabolic abnormalities were at elevated risk. However, in the subgroup analysis by physical activity, physically inactive MHO participants had a significantly higher HR for CVD events compared to active MHNO participants (HR, 1.54; 95% CI, 1.03–2.30), while active MHO participants were not at elevated risk (HR, 1.15; 95% CI, 0.70–1.89). Physically inactive MHO participants had significantly increased risk of CVD compared to physically active MHNO participants whereas physically active MHO participants did not.

## Introduction

The incidence of obesity is increasing and the worldwide prevalence of obesity more than doubled between 1980 and 2012 [1]. In Korea, the prevalence of obesity has increased over the past 10 years (20.6% to 24.6% in men, 16.2% to 17.3% in women from 2006 to 2015) [2]. These changes are particularly important given the known association between obesity with cardiovascular disease (CVD), diabetes, and death [3].

Intriguingly, obesity is not synonymous with metabolic disease, as people who are overweight or obese do not always have no metabolic dysfunction, especially cardio-metabolic dysfunction. These populations have been called the metabolically healthy obesity (MHO) phenotype. The underlying mechanisms which cause MHO phenotype among obese people are multiple and complex. This phenotype is the result of complex interaction between genetic, environmental, and behavioral factors [4]. The absence of genetic traits associated with fat distribution, reduced visceral fat mass and ectopic fat accumulation in liver, muscle, and pancreatic beta cells, and low systemic inflammation all contribute to metabolically healthy obesity [4].

The diagnostic criteria for MHO is controversial, and therefore the prevalence of MHO is difficult to quantify and varies according to the definition used [5]. The prevalence of MHO has been reported to be 10–25% worldwide [6, 7], while the estimated prevalence among middle-age men in Korea is 24.2–28.5% [8]. A recent review article reported that the prevalence of MHO showed a large variation from 6% to 75% [9].

Although, by definition, people with MHO are metabolically ‘healthy’, the metabolic outcomes of MHO remain unclear. Regarding cardiovascular outcomes in this group of patients, Hamer et al. [10] observed 22,203 subjects without CVD for more than 7 years. This observational study revealed that MHO did not increase the risk for all-cause or cardiovascular mortality. Furthermore, MHO has little association with heart failure [11] and with coronary atherosclerosis [12]. On the contrary, several large observational studies reported that MHO is a significant risk factor for CVD. Ogorodnikova et al. [13] conducted a 12-year observational study and demonstrated patients with MHO had high risk of CVD compared to metabolically healthy normal weight individuals. Kramer et al. [14] reviewed 8 studies about all-cause mortality and cardiovascular events and concluded that obesity is an independent risk factor for CVD in patients with and without metabolic disorders.

These discrepancies suggest that MHO alone is not sufficient to predict metabolic outcome and patients with MHO likely represent a heterogeneous group. Therefore, it remains important to identify the key factors that determine healthy outcomes in MHO and to re-define metabolically healthy obesity.

In this study, we focused on physical activity (PA) as a key factor, since it is well known to influence obesity, and because physical inactivity contributes to the development of metabolic disorders such as heart disease, hypertension, and type 2 diabetes mellitus (T2DM) [15]. The World Health Organization (WHO) recommends that adults should perform at least 150 minutes of moderate-intensity aerobic physical activity (PA) or 75 minutes of vigorous-intensity aerobic PA throughout the week [16]. The U.S. Department of Health and Human Services also recommends at least 150 minutes of PA for major health benefits [17].

Many studies have reported that the amount of PA has inverse dose-response associations with coronary heart disease and CVD [18] and regular exercise improves insulin sensitivity and reduces glycosylated hemoglobin (HbA1c) levels in T2DM patients [19].

In the present study, patients with MHO were divided into four groups based on their level of PA, and we evaluated the risk of CVD in a Korean population-based cohort. The aim of the present study was to determine the effect of PA on CVD outcomes of MHO and to re-define the concept of “real” metabolic health.

## Materials and methods

### Study population

This was a prospective cohort study in which data were collected from two population-based cohorts from Ansan and Ansung, Korea, and was as part of the Korean Genome and Epidemiology Study (KoGES), a Korean government-funded epidemiological survey to investigate trends in chronic noncommunicable diseases and associated risk factors. A total of 10,038 participants were examined at baseline in 2001–2002 and the biennial follow-up examination is ongoing. The design of the cohort study and the demographics of participants have been described elsewhere [20].

The 10-year follow-up data are included in the present study. The following participants were excluded from the present study: those with incomplete data (demographic, anthropometric, or laboratory), those with a clinical history of CVD or cancer at baseline, and those who had received steroids or anticoagulants. The total number of eligible participants was 8,144.

### Measurement

Height and body weight were measured to the nearest 0.1cm or 0.2kg, respectively. Blood pressure (BP) was measured in the sitting position after at least 5 minutes of rest. Blood samples were obtained after an overnight fast of at least 8 h, and biochemical assays including plasma glucose, total cholesterol (TC), triglycerides (TG), and high-density lipoprotein cholesterol (HDL-C) were measured using the ADVIA 1650 chemistry analyzer (Bayer HealthCare Ltd., Tarrytown, NY, USA). Hemoglobin A1c (HbA1c) level was measured using high-performance liquid chromatography (Variant II; BioRad Laboratories, Hercules, CA, USA)

The questionnaire included questions on sociodemographic information, lifestyle, personal and familial medical history, smoking status and alcohol consumption. The participants were classified as non-, ex-, and current drinkers and never-, ex-, and current smokers based on their response to lifetime alcohol consumption and smoking status on the questionnaire.

### Definition

There is no consensus regarding the diagnosis of metabolically healthy obesity. In the present study, non-obese participants were defined as those with a body mass index (BMI) of 18–25 kg/m^2^ and obesity as a BMI of ≥25 kg/m^2^ according to Asia-Pacific BMI criteria, which are defined by the WHO Western Pacific Region [21]. Metabolic risk was based on an adaptation of the Adult Treatment Panel-III (ATP-III) definition of metabolic syndrome, and defined as two or more metabolic risk factors, including hypertension (a systolic blood pressure (BP) at least 130 mmHg and/or a diastolic BP at least 85 mmHg, or on antihypertensive treatment), impaired fasting glucose (fasting plasma glucose at least 100 mg/dL), triglycerides (TG) at least 150 mg/dL, and adverse high density lipoprotein (HDL) cholesterol (1.03 mmol/L in men and 1.30 mmol/L in women). The waist circumference (WC) criterion was not used because of its collinearity with BMI. According to these criteria, participants were categorized into one of four groups consisting of metabolically healthy non-obese (MHNO); metabolically healthy obese (MHO); metabolically unhealthy non-obese (MUNO); and metabolically unhealthy obese (MUO). All participants completed a structured questionnaire that included health-related questions concerning PA. The PA categories included a physically active group (at least 30 minutes per day of PA) and an insufficient group (fewer than 30 minutes per day of PA). Because there was no data about average minutes of PA per week in the baseline data, we were unable to use the WHO recommendation [16]. However, the intensity and average minutes per week of PA were investigated at the first follow-up survey and we conducted subgroup analysis of participants with the first follow-up data using the WHO definition which recommends at least 150 minutes of moderate-intensity aerobic PA or 75 minutes of vigorous-intensity aerobic PA throughout the week. CVD events were investigated with a structured questionnaire and defined as any of the following: myocardial infarction, coronary heart disease, congestive heart failure, cerebrovascular disease, or peripheral arterial disease.

### Statistical analysis

All data are presented as the mean and standard deviation, or prevalence (%) and 95% confidence interval (CI). The independent sample t-test or one-way analysis of variance (ANOVA) was used to compare continuous variables and Pearson’s chi-square test was used to compare proportions according to metabolic health and obesity status. Cox proportional hazards regression models, unadjusted and adjusted, were constructed to evaluate the hazards ratio (HR) and 95% CI for CVD events in groups classified according to metabolic health and obesity status. Follow-up time was calculated as time from first anthropometric and clinical measures to date of CVD events or time from first anthropometric and clinical measurement to end of follow-up (December 31, 2012). Analyses were performed using SPSS version 21.0 software (IBM, Armonk, NY, USA). The significance levels were set at 0.05.

### Ethics statement

The institutional review board of Bundang CHA Hospital (South Korea) approved the study protocol (IRB No. CHAMC 2016-08-017), and all participants volunteered and provided written informed consent prior to their enrollment. All participants’ records were anonymized before being accessed by the authors.

## Results

### Baseline characteristics

Data from 8,144 participants (3,942 men and 4,202 women) were assessed. The anthropometric, clinical, and biochemical characteristics of the participants classified by obesity and metabolic health status are summarized in Table 1.

**Table 1:** Characteristics of subjects according to obesity and metabolic health status.

	MHNO (N = 2929)	MHO (N = 1262)	MUNO (N = 1743)	MUO (N = 2210)	P-value
Men, N	1501 (51.2%)	560 (44.4%)	875 (50.2%)	1006 (45.5%)	<0.001
Age, years	48 (43–58)	48 (43–56)	55 (46–63)	52 (45–60)	<0.001
Alcohol, N					<0.001
Never drinker	1265 (43.2%)	528 (41.8%)	819 (47.0%)	1102 (49.9%)	
Ex-drinker	167 (5.7%)	74 (5.9%)	116 (6.7%)	147 (6.7%)	
Current drinker	1497 (51.1%)	660 (52.3%)	808 (46.4%)	961 (43.5%)	
Smoking, N					<0.001
Never smoker	1651 (56.4%)	806 (63.9%)	979 (56.2%)	1342 (60.7%)	
Ex-smoker	434 (14.8%)	201 (15.9%)	283 (16.2%)	354 (16.0%)	
Current smoker	844 (28.8%)	255 (20.2%)	481 (27.6%)	514 (23.3%)	
Physical activity*, N	1139 (38.9%)	504 (39.9%)	616 (35.3%)	760 (34.4%)	0.001
BMI, Kg/m^2^	22.5 (21.0–23.7)	26.6 (25.7–28.0)	23.3 (22.0–24.2)	27.0 (26.0–28.7)	<0.001
Systolic BP, mmHg	112 (104–121)	114 (106–122)	127 (114–139)	128 (117–140)	<0.001
Diastolic BP, mmHg	75 (69–81)	78 (71–83)	84 (75–90)	86 (78–92)	<0.001
Total cholesterol, mg/dL	182 (161–205)	193 (169–217)	188 (165–213)	197 (175–222)	<0.001
HDL-C, mg/dL	48.0 (42.0–55.0)	46.0 (41.0–52.0)	40.0 (35.0–45.5)	39.0 (35.0–44.0)	<0.001
Triglycerides, mg/dL	104 (83–128)	114 (92–138)	180 (147–235)	189 (153–256)	<0.001
HbA1c, %	5.5 (5.3–5.7)	5.5 (5.3–5.8)	5.7 (5.4–6.0)	5.8 (5.5–6.2)	<0.001
Metabolic state, N					
Hyperglycemia	93 (3.2%)	47 (3.7%)	479 (27.5%)	687 (31.1%)	<0.001
Low HDL-C	876 (29.9%)	447 (35.4%)	1313 (75.3%)	1783 (80.7%)	<0.001
High TG	247 (8.4%)	163 (12.9%)	1290 (74.0%)	1700 (76.9%)	<0.001
CVD events^†^, N	135 (4.6%)	70 (5.5%)	155 (8.9%)	208 (9.4%)	<0.001
Follow up time, years	8.3 (8.2–8.4)	8.5 (8.3–8.6)	8.2 (8.0–8.3)	8.1 (8.0–8.3)	0.002

Abbreviations: MHNO, metabolically healthy nonobese; MHO, metabolically healthy obese; MUNO, metabolically unhealthy nonobese; and MUO, metabolically unhealthy obese; N; number, BP, blood pressure; HDL-C, high dense lipoprotein cholesterol, CVD, cardiovascular diseases.

* Participants who engaged in physical activity for at least 30 minutes per day

^†^ Participants who had either myocardial infarction, coronary heart disease, congestive heart failure, cerebrovascular disease or peripheral arterial disease

Data were presented as the means (95% CI) or numbers (%)

Among the included participants, 15.5% exhibited the MHO phenotype. Compared to MHNO participants, those with the MHO phenotype tended to be female and have a high BMI (both p<0.01). However, other characteristics such as mean age, alcohol drinking, smoking status, and metabolic parameters did not differ significantly between the two groups. Participants with MUNO and MUO phenotype were more likely to be older and to have higher BP, TG, and HbA1c levels, lower levels of HDL-C, and a higher prevalence of prehypertension and prediabetes than those with MHNO and MHO phenotypes.

### Association between CVD events and metabolically healthy-obesity phenotype

**Fig 1:**
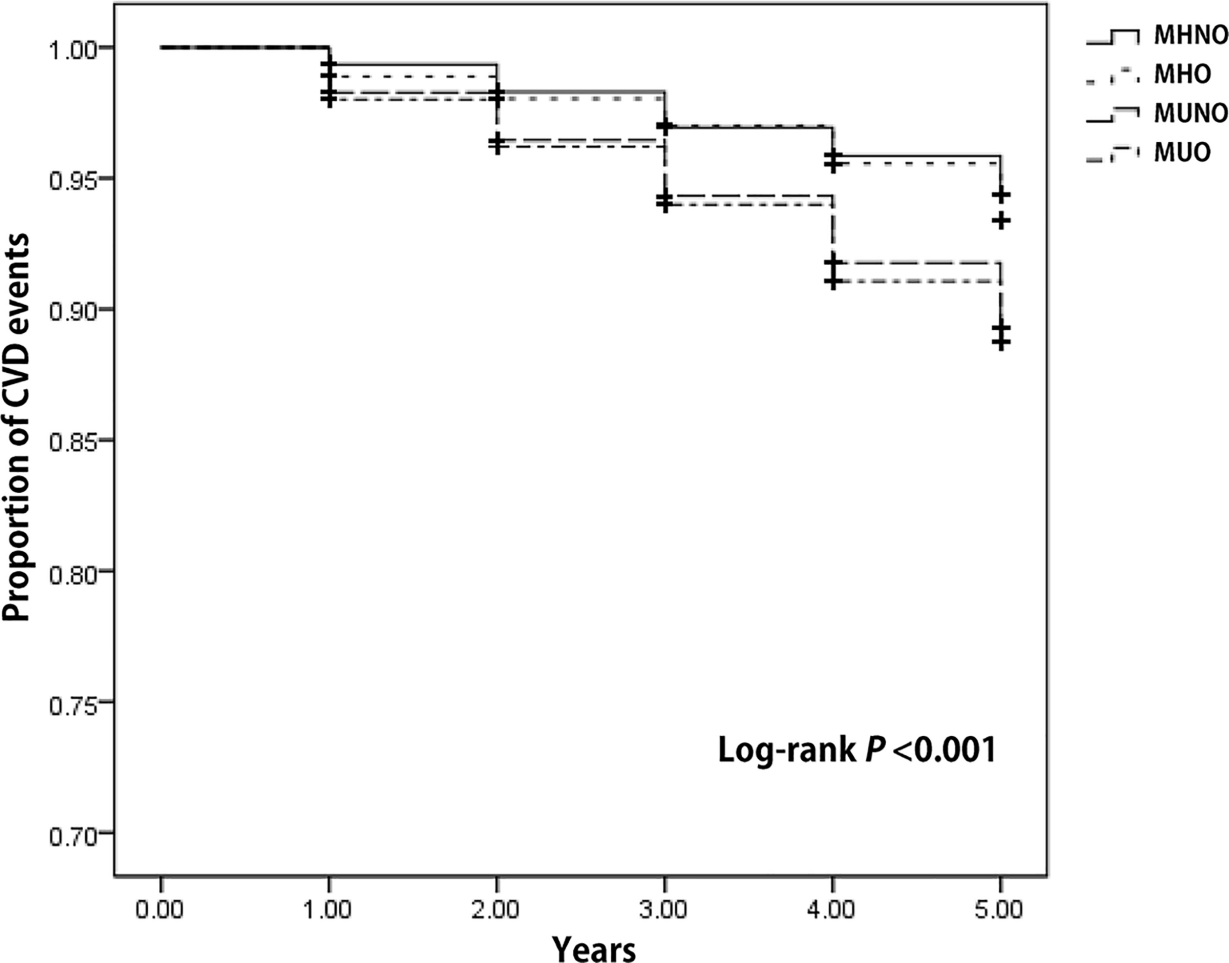
Kaplan–Meier curves of CVD events according to metabolic health and obesity status. During ten-year follow-up periods, the metabolically healthy group shows better event-free survival than the metabolic unhealthy group regardless of obesity.

A total of 568 CVD events were reported during the 10 years. Kaplan-Meier survival curves revealed a significantly higher rate of CVD events in participants with the MUNO and MUO phenotypes than in those with the MHNO phenotype (MUNO vs. MHNO, p < 0.001; MUO vs. MHNO, p< 0.001). However, there was no significant difference between participants with the MHNO and MHO phenotype (p = 0.272; Fig 1).

In a multivariate Cox regression model adjusted for age and gender, MHO participants were not at elevated risk of CVD compared with their MHNO counterparts (HR, 1.28; 95% CI, 0.96–1.71), although both the non-obese (HR, 1.50; 95% CI, 1.19–1.90) and obese (HR, 1.85; 95% CI, 1.48–2.30) participants with metabolic abnormalities were at elevated risk (Table 2). Similar results were seen after adjustment for age, gender, and other covariates such as alcohol drinking, smoking, PA, and medications.

**Table 2:** Hazard ratios for CVD events according to obesity and metabolic health status.

	Model1		Model2	
	HR (95%CI)	P	HR (95%CI)	P
MHNO	1 (Reference)		1 (Reference)	
MHO	1.280 (0.957–1.710)	0.096	1.288 (0.963–1.723)	0.088
MUNO	1.503 (1.190–1.899)	0.001	1.358 (1.071–1.724)	0.012
MUO	1.847 (1.484–2.299)	<0.001	1.596 (1.268–2.007)	<0.001

Abbreviations: MHNO, metabolically healthy nonobese; MHO, metabolically healthy obese; MUNO, metabolically unhealthy nonobese; and MUO, metabolically unhealthy obese; HR, hazard ratios; CI, confidential interval.

Model 1 was adjusted for age and gender.

Model 2 was adjusted for the variables in Model 1, plus alcohol drinking, smoking, physical activity and medication of hypertension, diabetes mellitus and dyslipidemia.

### Effects of physical activity on CVD events by obesity and metabolic health status

We further performed a subgroup analysis by PA status. Participants with the MUNO and MUO phenotype were more likely to be physically inactive than those with the MHNO and MHO phenotype (Table 1). The adjusted HRs of CVD events by PA, obesity, and metabolic health status are shown in Table 3.

**Table 3:** Hazard ratios for CVD events according to obesity, metabolic health status and physical activity.

	Model1		Model2	
	HR (95% CI)	P	HR (95% CI)	P
MHNO with active PA	1 (Reference)		1 (Reference)	
MHNO with inactive PA	1.129 (0.794–1.607)	0.498	1.112 (0.781–1.582)	0.557
MHO with active PA	1.147 (0.697–1.886)	0.590	1.147 (0.697–1.888)	0.590
MHO with inactive PA	1.536 (1.026–2.298)	0.037	1.524 (1.018–2.281)	0.041
MUNO with active PA	1.598 (1.081–2.361)	0.019	1.438 (0.970–2.133)	0.071
MUNO with inactive PA	1.640 (1.160–2.320)	0.005	1.467 (1.034–2.081)	0.032
MUO with active PA	1.890 (1.304–2.741)	0.001	1.608 (1.101–2.349)	0.014
MUO with inactive PA	2.051 (1.475–2.853)	<0.001	1.767 (1.262–2.474)	0.001

Abbreviations: MHNO, metabolically healthy nonobese; MHO, metabolically healthy obese; MUNO, metabolically unhealthy nonobese; and MUO, metabolically unhealthy obese; PA, physical activity; HR, hazard ratios; CI, confidential interval.

Model 1 was adjusted for age and gender.

Model 2 was adjusted for the variables in Model 1, plus drinking, smoking, physical activity and medication of hypertension, diabetes mellitus and dyslipidemia.

In a multivariate Cox regression model adjusted for age and gender, the HR for CVD events slightly increased from 1.89 (95% CI, 1.30–2.74) in active participants with MUO reaching 2.05 (95% CI, 1.48–2.85) in inactive counterparts with MUO compared to physically active MHNO participants. A similar increase of HR was found in those with MUNO as well (HR, 1.60; 95% CI, 1.08–2.36 in active participants; HR, 1.64; 95% CI, 1.16–2.32 in inactive participants). Compared to active MHNO participants, physically inactive MHO participants had a significantly higher HR for CVD events (HR, 1.54; 95% CI, 1.03–2.30), while active participants were not at elevated risk of CVD (HR, 1.15; 95% CI, 0.70–1.89). The subgroup analysis with the first follow-up data for PA showed comparable results (S1 Table). In addition, we conducted further analysis with MHNO and MHO participants by presence of metabolic abnormality and found that MHO with one metabolic abnormality and inactive PA increased the risk of CVD event (HR; 1.786; 95% CI, 1.047–2.987) whereas those with no metabolic abnormality or active PA did not (Table 4).

**Table 4:** Age and sex adjusted hazard ratios for CVD events according to obesity, metabolic health status and physical activity based on strict definition.

	HR (95% CI)	P
MHNO with active PA (no metabolic abnormality)	1 (Reference)	
MHNO with inactive PA (no metabolic abnormality)	1,115 (0.653–1.906)	0.690
MHO with active PA (no metabolic abnormality)	1.256 (0.536–2.945)	0.599
MHO with inactive PA (no metabolic abnormality)	0.950 (0.424–2.127)	0.900
MHO with active PA (one metabolic abnormality)	1.111 (0.584–2.113)	0.748
MHO with inactive PA (one metabolic abnormality)	1.768 (1.047–2.987)	0.033

Abbreviations: MHNO, metabolically healthy nonobese; MHO, metabolically healthy obese; MUNO, metabolically unhealthy nonobese; and MUO, metabolically unhealthy obese; PA, physical activity; HR, hazard ratios; CI, confidential interval.

## Discussion

In this study, we performed a long-term follow-up study of Korean patients and analyzed the risk for CVD in patients with MHO phenotype. The MUO and MUNO groups had significantly higher risk for CVD than the MHNO group. Although the present study failed to find significant results between the MHO and MHNO groups, the subgroup analysis by PA status showed that the MHO group with insufficient PA had worse prognosis than the MHNO group with sufficient PA. Interestingly, despite being obese, the prognosis of physically active individuals with MHO did not differ significantly from that of MHNO individuals.

These results show that metabolically healthy obese populations are not homogenous. This heterogeneity of ‘obesity’ is due to a limitation in the classification of obesity by BMI. First, obesity is defined as excess body fat mass, but BMI does not accurately reflect body fat mass [22], since bodyweight cannot distinguish between excess fat and excess muscle or bone mass. Second, extra fat is not always bad. Adipose tissue is a complex organ composed of white, beige, and brown fat. The beige/brown fat has many beneficial effects on metabolism [23]. Furthermore, the metabolic effects of fat differ according to its location. For example, excess visceral fat mass has deleterious effects on metabolism and is an independent risk factor of metabolic syndrome, type 2 diabetes, CVD and cancer [24, 25], whereas subcutaneous fat has beneficial effects on metabolism [26]. In one animal study, subcutaneous fat transplantation improved metabolic dysfunction in an obese mouse model [27].

In order to overcome this limitation of obesity classification according to BMI, many studies tried to evaluate obesity using other body index parameters such as waist circumference, waist hip ratio or waist height ratio [28, 29], and reported that these other indexes explained obesity related metabolic disorders than BMI. However, these indexes are still an indirect reflection of excess fat mass, and therefore additional clinical data accumulation is required to replace BMI. To measure regional body fat composition accurately, we can measure body composition or fat mass using computed tomography (CT), magnetic resonance imaging, DXA and PET-CT [30, 31]. Unfortunately, these methods are expensive, and have limited availability and accessibility for long-term follow up in a large-scale cohort study.

Therefore, the degree of PA is an easily measured and inexpensive alternative to the methods described above which is useful in predicting the CVD risk of obesity and a suitable alternative to overcome the limitation of BMI. In our data, the degree of PA acts an independent prognostic factor on the CVD outcome even in the same metabolic comorbidity. In fact, PA has been reported as a protective factor against the development of CVD in many clinical studies [18]. Jian Li et al. [32] reported that PA reduced the overall risk of CVD among men and women by 20–30% and 10–20% percent, respectively. PA is an independent regulator of body fat percent and body composition [33], and is associated with the activity of brown adipose tissue [34].

In our study cohort, physically active individuals comprised 39.9% of the MHO group (Table 1). More than half of the participants in the MHO group were physically inactive. When assessing the CVD outcome of the overall MHO group, this ratio resulted in an increased, but not statistically significant risk (HR 1.280, p> 0.05). Therefore, PA can provide a convincing explanation for the discrepant results of previous longitudinal studies about the risk of developing CVD in MHO [35–38]. Taken together, these findings demonstrate that the degree of PA should be considered alongside other determining factors such as obesity and metabolic comorbidity when predicting cardiovascular risk.

The main strength of this study is that this was a long-term population-based cohort study (10-year follow up) from a single ethnic group. This study also has several limitations. First, we were unable to analyze fat distribution or use other demographic indexes associated with obesity such as waist circumference. Therefore, we did not account for all of the important factors associated with CVD risk. Second, since we could not assess mortality data, we may have missed fatal CVD events.

In conclusion, we analyzed the CVD outcomes according to obesity and metabolic health status in a Korean population. Physically inactive MHO participants had significantly increased risk of CVD compared to physically active MHNO participants, whereas physically active MHO participants did not. Further studies are required to re-define MHO in view of PA status in order to better characterize ‘real’ metabolically healthy obesity.

## Supporting information

S1 Table Age and sex adjusted hazard ratios for CVD events according to obesity, metabolic health status and physical activity based on the WHO definition. Abbreviations: MHNO, metabolically healthy nonobese; MHO, metabolically healthy obese; MUNO, metabolically unhealthy nonobese; and MUO, metabolically unhealthy obese; PA, physical activity; HR, hazard ratios; CI, confidential interval.
(PDF)
