# Supplementary material for: The influence of physical activity on risk of cardiovascular disease in people who are obese but metabolically healthy
Source: PLoS One. 2017 Sep 26;12(9):e0185127. doi: 10.1371/journal.pone.0185127 (PMC5614526; doi:10.1371/journal.pone.0185127)
Supplement: S1 Table — Abbreviations: MHNO, metabolically healthy nonobese; MHO, metabolically healthy obese; MUNO, metabolically unhealthy nonobese; and MUO, metabolically unhealthy obese; PA, physical activity; HR, hazard ratios; CI, confidential interval. (PDF) [file pone.0185127.s001.pdf]

S1 Table. Age and sex adjusted hazard ratios for CVD events according to obesity, metabolic health status and physical activity based on the WHO definition.

	HR (95% CI)	P
MHNO with active PA	1 (Reference)	
MHNO with inactive PA	1.454 (0.951-2.224)	0.084
MHO with active PA	1.552 (0.884-2.726)	0.126
MHO with inactive PA	1.822 (1.134-2.928)	0.013
MUNO with active PA	2.335 (1.455-3.748)	<0.001
MUNO with inactive PA	1.880 (1.223-2.889)	0.004
MUO with active PA	2.215 (1.405-3.491)	0.001
MUO with inactive PA	2.548 (1.682-3.862)	<0.001

Abbreviations: MHNO, metabolically healthy nonobese; MHO, metabolically healthy obese; MUNO, metabolically unhealthy nonobese; and MUO, metabolically unhealthy obese; PA, physical activity; HR, hazard ratios; CI, confidential interval.
